# Advances in Fish Gene Editing

**DOI:** 10.3390/ani16121874

**Published:** 2026-06-17

**Authors:** Jiaqing Xu, Fangzhou Cheng, Junchao Fang, Kun Cao, Guanglve Li, Wenyin Luo, Dan Hu, Junjie Zhang, Qiaomu Hu

**Affiliations:** 1 State Key Laboratory of Mariculture Biobreeding and Sustainable Goods, Yellow Sea Fisheries Research Institute, Chinese Academy of Fishery Sciences, No. 106 Nanjing Road, Qingdao 266071, China; x320243911@163.com (J.X.); 13245460338@163.com (F.C.); 15119438380@163.com (J.F.); 13659644863@163.com (G.L.); luowenyin7235@163.com (W.L.); danhu1001@163.com (D.H.); 2 College of Life Sciences, Xinjiang Agricultural University, Urumqi 830052, China; 3 Hainan Fisheries Innovation Research Institute, Chinese Academy of Fishery Sciences, Sanya 572024, China; caokun618@163.com; 4 Yazhoubay Agriculture and Aquaculture Development Co., Ltd., Sanya 572025, China

**Keywords:** gene editing, gene editing tools, delivery strategies, aquaculture breeding, biosafety

## Abstract

Fish represent the most species-rich group within the phylum Chordata, possessing exceptional nutritional and ornamental value. Global aquaculture, particularly finfish farming, is experiencing rapid expansion worldwide, and fish serve as crucial model organisms for vertebrate developmental biology and functional genomics research. However, traditional breeding methods are plagued by limitations such as low precision and lengthy breeding cycles. Currently, gene editing technologies represented by the CRISPR/Cas system, base editing, and prime editing have provided revolutionary tools for dissecting gene function, modeling human diseases, targeted trait improvement, and ecological adaptation studies. This review describes the evolutionary history of gene editing technology, compares gene delivery strategies in fish embryos, and highlights landmark applications in key areas, including gene function research, aquaculture breeding, ornamental fish coloration regulation, and human disease model construction. Finally, we propose that innovation should be pursued while ensuring biosafety and regulatory compliance, to promote the transformation of fish gene editing toward large-scale and safe application.

## Simple Summary

This comprehensive review provides a thorough overview of the evolutionary trajectory of gene editing technologies and their applications and breakthroughs in fish at each developmental stage. It places particular emphasis on recently developed novel gene editors and the strategies for delivering gene editing tools into fish embryos. Furthermore, it elucidates the major breakthroughs achieved by gene editing technologies in four key domains. Finally, it clearly articulates that technological innovation should continue to be advanced under the premise of strictly ensuring biosafety and complying with relevant regulations, enabling the safer and larger-scale application of fish gene editing technologies in practical production and scientific research.

## 1. Introduction

Owing to their unique biological characteristics, fishes offer multiple compelling advantages as research organisms for gene editing studies. The early-stage embryos of model fish species such as zebrafish and medaka are highly transparent and develop through external fertilization, enabling non-invasive in vivo imaging [1], cell tracing, and phenotypic profiling without inflicting embryonic damage. Furthermore, delivery manipulations including microinjection and electroporation can be conducted during the early cleavage stages post-fertilization to generate germline-transmissible mutants. These aforementioned advantages establish them as ideal model organisms for vertebrate genetic research. Such studies have not only elucidated in depth the molecular mechanisms of gene action but also served as invaluable experimental platforms for investigating human genetic disorders [2,3,4].

Fishes exhibit high fecundity and short, rapid reproductive cycles, which represent one of the key factors facilitating accelerated gene function research, multi-generational genetic validation, and high-throughput screening. Taking zebrafish as a paradigmatic example, it reproduces rapidly, producing hundreds of eggs per week with a generation time of merely 3–4 months. This characteristic significantly accelerates the validation of gene editing efficacy and multi-generational selection, enabling multi-generational genetic analyses and long-term tracking of phenotypic stability. Furthermore, fishes possess extensive repositories of transgenic lines and CRISPR/Cas9-based toolkits, which support large-scale screening of target genes from CRISPR libraries and precise gene knockout or knock-in, thereby facilitating functional genomics investigations [5]. In contrast to mammalian models, fishes serve as highly cost-effective experimental subjects that are amenable to large-scale husbandry. They can be reared at high densities in small-scale aquatic systems, which greatly facilitates high-throughput gene editing screens.

Fishes exhibit a unique combination of genomic conservation and diversity, sharing extensive sets of orthologous genes and conserved signaling pathways with humans. This enables cross-species extrapolation of biological mechanisms across multiple domains including development, immunology, neurobehavior, and metabolism, thus rendering them widely utilized for human disease modeling and drug screening. Fishes share a substantial repertoire of orthologous genes with mammals. The Wellcome Sanger Institute has generated the high-quality reference genome of zebrafish, based on which subsequent comparative genomic analyses between zebrafish and human genomes have revealed that approximately 70% of human genes have at least one identifiable zebrafish ortholog [6,7]. This high degree of genomic conservation underscores the remarkable similarities between fishes and humans in organ development and immune system function, facilitating gene editing-based investigations into neurodegenerative diseases and offering novel diagnostic and therapeutic strategies for psychiatric disorders [8]. Previously, several identified genes involved in the regulation of the Wnt/β-catenin signaling pathway have been implicated as risk factors for human psychiatric disorders [9]. For instance, the majority of Cornelia de Lange syndrome (CdLS) cases are caused by haploinsufficiency of the *nipbl* gene. Kawauchi et al. [10] employed molecular genetic tools to develop both mouse and zebrafish models of CdLS, generating *nipbl*-deficient mouse and zebrafish lines.

At the fundamental research level, gene editing technologies have transformed the conclusion that manipulating specific genes can alter corresponding phenotypes from correlative associations into reproducible and verifiable functional evidence. Targeted gene knockout or knock-in enables researchers to elucidate the molecular basis underlying fish development, reproduction, and evolution, unravel gene functions and the regulatory mechanisms of biological processes, thereby providing robust support for basic scientific investigations [11]. At the industrial level, gene editing technologies enable targeted modification of growth-related genes and immune-associated genomic loci, facilitating the rapid development of high-yield and stress-resistant aquaculture varieties. This approach drastically shortens the generation time required for conventional selective breeding and enhances both overall aquaculture productivity and feed conversion efficiency (FCE) [12]. In the field of disease research, gene editing technologies—through targeted gene knockout, knock-in, and precise mutagenesis—enable researchers to elucidate the molecular mechanisms underlying development and organogenesis, identify key regulatory nodes in immune responses, and generate fish models harboring human pathogenic genes for mechanistic investigations and high-throughput drug screening. These versatile models have broad applications in both human translational medicine and aquaculture health management: by engineering fish genomes to recapitulate human genetic disorders or the pathogenesis of aquatic pathogens, they facilitate the discovery of novel therapeutic targets and strategies, as well as the enhancement of disease resistance in farmed fish species [13]. However, gene editing technologies also raise significant concerns across biosafety, ethical, and regulatory domains. Their development must proceed in tandem with robust risk assessment frameworks to advance sustainable aquaculture and environmental protection. Specifically, these technologies enable the development of environmentally benign transgenic fish, implementation of reproductive containment strategies to prevent ecological invasion, and optimization of nutritional traits to reduce resource consumption [14].

Taken together, fishes represent an exceptionally amenable system for gene editing research, and investigations into fish gene editing hold profound scientific and industrial significance. Early site-specific genome modification relied primarily on homologous recombination (HR), which exhibited extremely low efficiency in multicellular organisms. Following the advent of the programmable nuclease era, zinc-finger nucleases (ZFNs) and transcription activator-like effector nucleases (TALENs) recognized target DNA sequences through their protein domains and induced double-strand breaks (DSBs) via the FokI endonuclease, markedly enhancing the feasibility of targeted genomic engineering. Subsequently, CRISPR/Cas systems, operating through an RNA-guided mechanism, enabled simpler and more flexible target design as well as multiplex editing, thereby becoming the most widely adopted gene editing platform to date. This review summarizes the evolutionary trajectory of gene editing tools, compares the strategies, efficiencies, and applicability of various delivery methods in fish embryos, highlights advances in delivery approaches and novel editing technologies, and concludes with their landmark applications in fish gene function research, aquaculture breeding, coloration enhancement in ornamental fish, and human disease modeling.

## 2. Evolution and Technical Essentials of Gene Editing Tools

### 2.1. Technological Milestones: From Homologous Recombination to Programmable Nucleases

The emergence of restriction endonucleases and homologous recombination marked the formal inception of gene editing technologies. These techniques enabled precise DNA replacement but exhibited inherently low efficiency, and were primarily applied to model organisms such as yeast and mice. Although they were not utilized in fish species at that time, they nonetheless represent the foundational origins of all subsequent gene editing methodologies. Scherer and Davis [15] developed a potentially universal method in 1979 that enabled the stable integration of in vitro-constructed exogenous sequences or deletion mutations into the chromosomes of *Saccharomyces cerevisiae*. Utilizing the *ura3* gene as a selectable marker, this method successfully achieved two key objectives: generating an internal deletion mutant of the *his3* gene and transposing a galactose-inducible region to chromosome XV, with all resulting engineered strains being completely devoid of any vector sequences. In 1985, Smithies et al. [16] developed a specialized plasmid carrying the *globin* gene through a series of experimental procedures including identification, cell culture, screening, and cloning. Following the introduction of this plasmid into mammalian cells and its subsequent integration into the human β-globin locus, the plasmid-borne gene sequence could undergo homologous recombination with the corresponding endogenous chromosomal sequence, ultimately achieving the intended targeted genetic modification in transformed cells at a frequency of approximately 1 in 1000. In 1986, Thomas KR et al. [17] demonstrated the correction of defective genes on mammalian cell chromosomes via microinjection. They injected copies of the gene carrying distinct mutations into cell nuclei and systematically investigated how gene targeting frequency was modulated by multiple factors, including the number of injected molecules, as well as the copy number, arrangement, and chromosomal localization of integrated genes, ultimately achieving successful targeted gene correction.

The discovery and development of zinc-finger nucleases marked the dawn of the programmable nuclease era. Zinc finger proteins (ZFPs) were first identified in the transcription factor IIIA of *Xenopus laevis* in 1983 [18], while zinc-finger nucleases (ZFNs) were first investigated and applied in the late 1990s [19]. As the first generation of programmable gene editing tools, ZFNs are fusion proteins composed of zinc-finger protein domains responsible for DNA sequence recognition and FokI nuclease cleavage domains [18], and were primarily utilized for genomic modification in mammalian cells. Each individual zinc-finger module recognizes 3–4 base pairs (bp), and modular assembly of these modules enables precise targeting of specific genomic loci. FokI nucleases require dimerization to exert their DNA cleavage activity, which generates DSBs. These DSBs subsequently trigger the cellular non-homologous end joining (NHEJ) or homology-directed repair (HDR) pathways, thereby mediating targeted gene knockout, knock-in, or correction—a technological advance of epoch-making significance. Pavletich et al. [20] determined the crystal structure of the complex formed by the three zinc-finger domains of Zif268, a mouse immediate early protein, and its consensus DNA binding site. This landmark study elucidated the molecular mechanism underlying zinc-finger-mediated DNA recognition. This discovery not only advanced our theoretical understanding of this mechanism but also provided the structural foundation for the rational design of novel DNA-binding proteins. Rouet et al. [21] were the first to employ the I-SceI meganuclease system to generate site-specific double-strand breaks in mouse chromosomes. This technological breakthrough laid a robust foundation for in-depth investigations into key cellular mechanisms, including DSB repair and homologous recombination, and ultimately revolutionized the entire landscape of mammalian genome analysis methodologies. In fish species, Meng et al. [22] designed ZFNs targeting the kdra gene in zebrafish. By microinjecting mRNA encoding these ZFNs into zebrafish embryos, they successfully induced targeted mutagenic lesions at the intended genomic loci. Doyon et al. [23] independently engineered ZFNs targeting the zebrafish *ntl* gene. Using the same mRNA microinjection strategy, they obtained a high proportion of animals carrying the desired gene mutations and exhibiting the expected loss-of-function phenotypes. Published simultaneously, these two landmark studies achieved heritable targeted gene knockout in fish for the first time, resolving the long-standing challenge of site-specific genetic manipulation in zebrafish at that time. This pivotal breakthrough laid the essential foundation for the subsequent widespread application of gene editing technologies across diverse fish species.

The advent of transcription activator-like effector nucleases (TALENs) rendered gene editing technology more straightforward and endowed it with superior specificity, while greatly expanding the range of applicable species and enabling its widespread deployment in both fish and plants. Moscou and Bogdanove [24] deciphered the recognition code of TAL effector proteins from *Xanthomonas* bacteria: highly specific DNA binding is achieved through repeated amino acid modules, with each module recognizing a single nucleotide. Each TALEN unit consists of a TAL recognition domain and a FokI cleavage domain, which must function in pairs to generate DSBs at target loci, facilitating subsequent DNA repair pathways. The discovery of this novel protein-DNA recognition mechanism comprehensively explained the targeting specificity of TAL effector proteins, enabled precise prediction of target sites, and laid a solid foundation for their scientific research and biotechnological applications. However, TALENs have limitations that restrict their large-scale application: their relatively large protein size causes delivery difficulties, and their construction still requires customized synthesis [25].

In fish research, Sander et al. [26] designed TALENs targeting the *kdr* and *ntl* genes in zebrafish. Through microinjection of mRNA into zebrafish embryos, they successfully induced high-frequency targeted mutations in somatic cells with mutation efficiencies ranging from 40% to 80%. Furthermore, Huang et al. [27] designed TALENs targeting the zebrafish *tnikb* and *dip2a* genes. They not only successfully induced mutations in somatic cells but also, importantly, demonstrated for the first time that TALEN-induced mutations could be stably transmitted to offspring via the germline. This represented the first achievement of heritable gene editing using TALEN technology in vertebrates. Cui et al. [28] broke through the technical bottleneck of embryonic microinjection in marine flatfish and successfully applied TALEN technology to a marine aquaculture fish species, the half-smooth tongue sole (*Cynoglossus semilaevis*), for the first time. By targeted knockout of the *dmrt1* gene on the Z chromosome, they demonstrated that *dmrt1* is the male-determining gene in half-smooth tongue sole and found that male fish with *dmrt1* mutations exhibited significantly accelerated growth rates. This was the first international study to successfully achieve gene editing and validate gene function in a marine fish species.

The success of these studies collectively demonstrates that as gene editing tools continue to improve, an increasing number of research questions are being addressed. This has gradually refined our understanding of fish life sciences and provides strategic support for the development of the Blue Granary. 

### 2.2. Working Mechanism and Applications of the CRISPR/Cas9 System

First identified by Ishino et al. in 1987 in the region adjacent to the *alkaline phosphatase* gene of *Escherichia coli* K12 [29], the structure later designated as CRISPR (Clustered Regularly Interspaced Short Palindromic Repeats) was formally named based on its characteristic sequence organization [30]. The CRISPR-Cas9 system originates from the adaptive immune system of bacteria and archaea, which provides defense against invading viruses and plasmids. In 2012, Jinek et al. [31] elucidated the molecular mechanism by which CRISPR RNA (crRNA) guides the recognition and cleavage of foreign nucleic acids. Mature crRNA forms a dual-RNA complex with trans-activating crRNA (tracrRNA) through complementary base pairing. This complex, often engineered as a single guide RNA (gRNA), directs the Cas9 endonuclease to specific genomic loci. The gRNA recognizes the target sequence via Watson–Crick base pairing, and Cas9 generates DSBs in the DNA immediately adjacent to a protospacer adjacent motif (PAM), typically the sequence NGG. These DSBs are subsequently repaired by endogenous cellular DNA repair pathways, which drive the desired gene editing outcomes.

Following this seminal study, the CRISPR/Cas system rapidly gained widespread adoption. Owing to its simplicity, cost-effectiveness, and capability for multiplex gene editing, it has become the dominant gene editing platform, with applications spanning from bacteria and archaea to mammalian cells, fish, and human cells. However, early versions of the system suffered from several limitations, including relatively high off-target risk, strict PAM sequence dependence, and potential immunogenicity concerns [32]. Subsequent optimization efforts have focused on enhancing precision, reducing off-target effects, and improving delivery efficiency. Major advancements include the development of high-fidelity Cas variants, paired Cas9 nickase systems for staggered DNA cleavage, transient expression via ribonucleoprotein (RNP) delivery, and refined gRNA design algorithms combined with improved off-target prediction tools. Hwang et al. [33] first demonstrated that Streptococcus pyogenes Cas9 (SpCas9) could efficiently induce targeted gene mutations in zebrafish embryos. They established a simple and rapid CRISPR/Cas9 gene editing protocol for zebrafish, laying the essential foundation for the subsequent widespread application of this technology across diverse fish species. Furthermore, Chang et al. [34] performed targeted knockout of the etsrp, gata4, and gata5 genes in zebrafish, successfully inducing biallelic mutations. Notably, they achieved, for the first time, the site-specific integration of exogenous DNA fragments in zebrafish.

### 2.3. Precision Editing: Base Editing, Prime Editing, and Epigenetic Regulation

To mitigate the risks of large-scale indels and chromosomal rearrangements caused by DSBs, gene editing technologies have progressively evolved toward DSB-free precision gene editing strategies. Base editing achieves single-base conversions such as C-to-T or A-to-G through the fusion of catalytically impaired Cas variants with deaminase domains. Operating without inducing DSBs, this technology significantly reduces the risk of unintended insertions and deletions. This DSB-independent mechanism makes base editing particularly suitable for the direct introduction or reversion of single-nucleotide variants (SNVs), with broad applications in modeling human pathogenic mutations, validating the function of conserved genomic loci, and generating allelic series. Key technical considerations for base editing include: the editing window (the relatively fixed range of bases within the gRNA target sequence that are susceptible to modification), bystander edits (unintended editing of adjacent bases within the same window), and genome-wide or transcriptome-wide off-target noise resulting from promiscuous deamination activity. In practice, these limitations can be mitigated by prioritizing the use of engineered high-fidelity base editors, minimizing cellular exposure time to editing components, and conducting systematic validation of both on-target editing outcomes and potential off-target sites using high-depth next-generation sequencing. The characteristics and comparative evaluation of different gene editing platforms and derivative technologies are presented in Table 1.

To date, most successful applications of base editing in fish have been achieved in the model organism zebrafish (*Danio rerio*). Cornean et al. [52] employed adenine and cytosine base editors to target the *dapk3*, *ube2b*, *usp44*, and *ptpn11* genes, modeling congenital heart disease in zebrafish. They validated the function of specific SNVs and established robust genotype-phenotype correlations in both F0 and F_1_ generations, demonstrating the practical value of base editing for disease gene functional studies. Furthermore, Qin et al. [53] developed the ABE-ultramax system, which achieved highly efficient biallelic adenine base editing in live zebrafish. This system outperformed standard base editors and has been applied to studies of developmental signaling and human pathology. Notably, Raudstein et al. [54] introduced precision single-base editing in Atlantic salmon (Salmo salar) embryos using the AncBE4max editor, achieving efficient C-to-T conversions. This technology enables functional analysis of genomic loci to enhance disease resistance potential in aquaculture, and the results demonstrated that AncBE4max provides a straightforward and highly effective approach for precise single-nucleotide editing in the Atlantic salmon genome. Additionally, Rosello et al. [55] successfully engineered a novel base editor capable of recognizing the NAA protospacer adjacent motif (PAM). They introduced C-to-T point mutations in the zebrafish *ctnnb1* gene, accurately recapitulating human oncogenic mutations and reproducing the resulting constitutive activation phenotype of the Wnt signaling pathway. They also performed targeted editing of cancer-related genes, such as cbl, and successfully generated a novel zebrafish dwarfism model. This study significantly advanced the genetic manipulation capabilities of zebrafish models, enabling more precise modeling of human disease mutations and providing more powerful tools for investigating endogenous signaling pathways and modeling human genetic disorders.

Prime editing fuses a Cas9 nickase with a reverse transcriptase and enables more flexible substitutions, insertions, and deletions under the guidance of prime editing guide RNAs (pegRNAs), without the requirement for donor DNA. This technology can achieve various types of precise substitutions, insertions, and small deletions, theoretically overcoming the limitations imposed by proximity to PAM sequences and the restricted editing windows of base editors. Qin et al. [56] optimized prime editing efficiency and introduced targeted mutations in zebrafish (Danio rerio). By forming ribonucleoprotein (RNP) complexes with the PE7 system and La-pegRNA and microinjecting them into embryos, they achieved editing efficiencies as high as 15.99%. Specifically, they introduced the P302L mutation in the *tyr* gene, which resulted in reduced melanin pigmentation. Furthermore, Petri et al. [57] utilized RNP complexes for prime editing delivery in zebrafish, enabling precise DNA insertions and substitutions for gene functional analysis. This delivery approach outperformed traditional methods using DNA or mRNA vectors in terms of editing efficiency.

**Figure 1:**
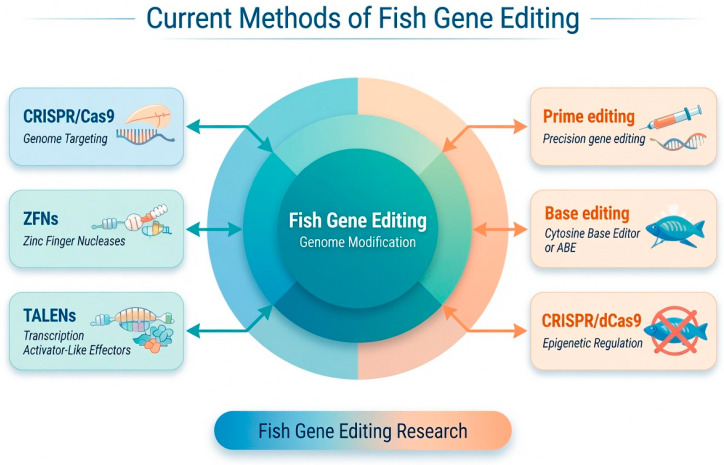
Current Methods for Gene Editing in Fish.

Unlike conventional gene editing that results in permanent genomic sequence alterations, dCas9 fused to transcriptional activation/repression domains or methylation/demethylation enzymes enables reversible gene regulation without modifying the underlying DNA sequence. This unique feature makes epigenetic editing particularly suitable for dissecting critical developmental windows, environmental stress responses, and phenotypic plasticity. Compared to gene knockout strategies, this approach more closely mimics dosage effect studies. It can be integrated with multi-omics technologies, including transcriptomics, chromatin accessibility profiling, and methylomics to construct comprehensive gene regulatory networks and identify potential molecular targets for aquaculture breeding. Liang et al. [58] demonstrated that CRISPR/dCas9-Dnmt7 and -Tet2 systems exhibit considerable potential as efficient tools for site-specific DNA methylation editing in zebrafish models. Chong-Morrison et al. [59] employed an engineered maize Ac/Ds transposon system to achieve high-efficiency identification of cis-regulatory elements in F0 zebrafish embryos and, through stable expression of guide RNAs, established a CRISPR/dCas9 enhancer perturbation technique (CRISPRi) that preserves the native DNA sequence. This work underscores the potential of the Ac/Ds system as a transient epigenetic regulatory tool in the zebrafish model. A schematic summary of current gene editing methods is presented in Figure 1.

### 2.4. Delivery Methods for Gene Editing in Fish

**Figure 2:**
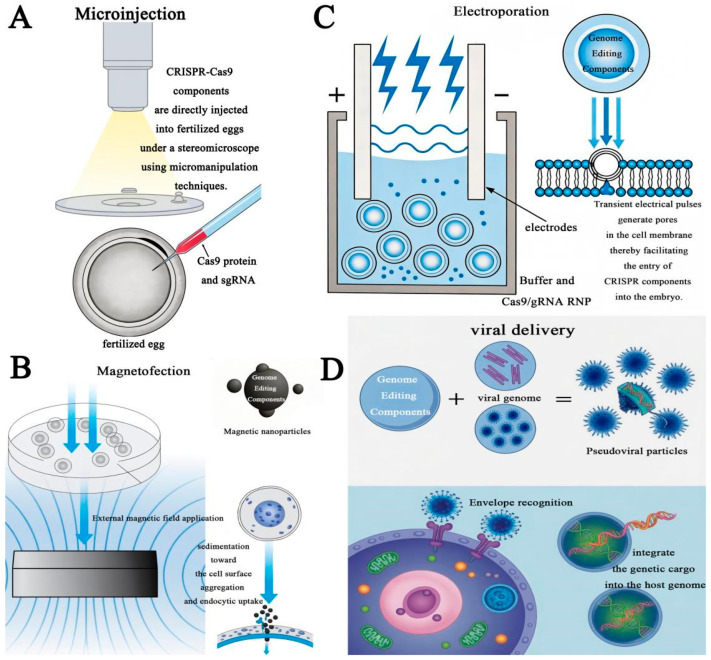
Delivery Methods for Gene Editing in Fish. (**A**) Microinjection: CRISPR-Cas9 components are directly injected into fertilized eggs at the 1–8 cell stage under a stereomicroscope via micromanipulation. (**B**) Magnetofection: Magnetic nanoparticle-coupled editing complexes are driven by an external magnetic field to aggregate on the cell surface and enter cells via endocytosis. (**C**) Electroporation: Transient electrical pulses generated by electrodes create reversible pores in the cell membrane, facilitating the entry of Cas9/gRNA ribonucleoprotein complexes and other CRISPR components into embryos. (**D**) Viral delivery: Pseudoviral particles carrying editing cassettes recognize cell surface receptors via envelope proteins and deliver the genetic cargo into target cells, with certain viral vectors integrating into the host genome.

Delivery methods for fish gene editing primarily target embryos or cells, aiming to deliver editing system components including single guide RNA (sgRNA) to target genomic loci. They are generally classified into three major categories: physical, chemical, and biological delivery approaches. All these methods must take into account the unique physiological characteristics of fish, such as their aquatic environment and rapid embryonic development. Physical methods rely on mechanical or electrical forces to directly penetrate cell membranes. Chemical methods utilize nanocarriers to encapsulate editing components, thereby enhancing their stability. Biological methods harness the high infectivity of viral vectors, but require careful control of host immune responses. A schematic diagram illustrating the mechanisms of delivery methods for fish gene editing is shown in Figure 2.

The microinjection method usually involves injecting into the cytoplasm of early cells. The 1–4 cell division stage is the early cell development period, during which the injection works best, and it can be extended as late as the 8-cell stage. This method allows researchers to flexibly adjust the dosage of CRISPR components and precisely deliver them to fertilized egg cells, ensuring efficient germline editing. However, mechanical damage exerts a certain impact on embryo survival rates, and editing efficiencies are highly variable post-injection. To address these limitations, fluorescently labeled Cas9 proteins can be employed for real-time tracking of delivery efficiency. Edvardsen et al. [60] were the first to successfully apply CRISPR/Cas9 technology to marine cold-water species. They achieved targeted knockout of two pigment biosynthesis-related genes, tyrosinase and solute carrier family 45 member 2 (*slc45a2*), in Atlantic salmon (*Salmo salar*). The study demonstrated that targeted biallelic mutations could be obtained using this technology despite the presence of mosaicism. Fangzhou et al. [61] have for the first time successfully applied CRISPR/Cas9 technology to edit the *cyp19a1a* gene in the tiger grouper (*Epinephelus fuscoguttatus*) and established an indoor rearing system for genome-edited juveniles. Five individuals were confirmed to harbor successful *cyp19a1a* knockout mutations via fin-clipped genotyping at 60 dpf. Elaswad et al. [62] developed a direct microinjection protocol for CRISPR/Cas9 protein delivery in channel catfish (*Ictalurus punctatus*). By targeting the *TICAM1* and *RBL* genes, they successfully achieved gene knockout, and sequencing confirmed the presence of insertion/deletion mutations at the edited loci, thereby establishing a more efficient method for gene disruption in this species. Li et al. [63] developed and validated a CRISPR/Cas9 gene editing system applicable to non-traditional aquatic organisms such as cichlids. Using pigmentation loss resulting from tyrosinase gene knockout as a visual readout, they established a rapid and reliable method for evaluating editing efficiency and concurrently overcame technical barriers associated with subcutaneous imaging, enabling the in situ visualization of internal structures and fluorescent signals.

The electroporation method utilizes transient electrical pulses to generate transient pores in the cell membrane, thereby facilitating the entry of CRISPR components into embryos or tissues. For fish embryos, electrical parameters, buffer composition, and developmental stage windows significantly influence both survival rates and editing efficiency, necessitating systematic optimization tailored to species and embryo size. This approach enables simultaneous processing of large numbers of diverse samples; however, it may induce substantial cellular damage and potentially compromise embryonic viability. Zhang et al. [64] established an electroporation protocol for zebrafish embryos, achieving efficient delivery of various genetic tools—including plasmid DNA, Cas9 protein, and guide RNAs—through systematic adjustment of square-wave electroporation parameters. Jin et al. [65] employed electroporation to deliver the CRISPR/Cas9 system into embryos of the Fujian oyster (Crassostrea angulata), detecting single-nucleotide substitution mutations in D-shaped larvae, thereby providing a reference for further application of electroporation in mollusks. Although oysters are mollusks, they can provide methodological references and value for gene editing of fish and even aquatic organisms, demonstrating the potential of electroporation delivery methods and the possibility of widespread application in the future. Zoppo et al. [66] delivered ribonucleoprotein (RNP) complexes composed of Cas9 protein and fluorescently labeled crRNA/tracrRNA targeting the *cyp1a1* gene via electroporation into rainbow trout (*Oncorhynchus mykiss*) intestinal cells, achieving an overall gene editing efficiency of 39%.

Viral delivery involves inserting the Cas9 protein and gRNA into the viral genome, followed by packaging of viral particles in helper cells to generate pseudoviruses. Upon transduction of target fish cells, the viral envelope recognizes cell surface receptors and delivers the genetic cargo into fish cells or embryos. During this process, vectors such as lentiviruses can integrate the genetic material into the host genome, enabling stable expression of Cas9 and gRNA. The Cas9-gRNA complex is subsequently guided to the target gene locus, where it induces DNA DSBs. Gene editing is achieved through either NHEJ or HDR. Gratacap et al. [67] used lentiviral delivery of CRISPR/Cas9 to knock out the *RIG-I* gene in the Chinook salmon (*Oncorhynchus tshawytscha*) cell line CHSE-214, which enhanced the efficiency of viral resistance studies.

Magnetofection is a promising non-viral delivery method that has shown high efficiency in certain mammalian and fish cell lines. It utilizes complexes composed of magnetic nanoparticles and nucleic acids or gene editing components, which, under the action of an external magnetic field, accelerate sedimentation and aggregation of the complexes on the cell surface and promote endocytic uptake [68], thereby significantly improving delivery efficiency. Its principle is based on magnetic force driving: the magnetic field can rapidly pull the complexes toward target cells, reducing diffusional losses and simultaneously lowering the required dosage. This method offers advantages including high transfection efficiency [69], short processing time, low cytotoxicity, and spatially targeted control [70], making it particularly suitable for hard-to-transfect primary cells or certain aquatic organism cells. In recent years, it has been extensively explored for the delivery of gene editing tools such as CRISPR/Cas9. By optimizing nanoparticle coatings to further enhance editing efficiency, progress has been made in fields including mammalian cells and plants [71,72,73], serving as an important complement to non-viral delivery systems.

In fish research, Arana et al. [74] compared three CRISPR/Cas9 delivery strategies—electroporation, lipid nanoparticles (LNPs), and magnetofection—for editing the *ifi27l2a* gene in DLB-1 (brain cells from European sea bass, *Dicentrarchus labrax*) and SaB-1 (brain cells from gilthead sea bream, *Sparus aurata*) cell lines. The results showed that magnetofection achieved high cellular uptake and good colocalization; however, no detectable gene editing was observed. This study highlights the cellular uptake advantages of magnetofection in fish cells and provides a novel framework and additional practical directions for gene editing approaches in future aquaculture.

## 3. Application of Gene Editing in Fish

### 3.1. Functional Studies of Fish Genes

CRISPR-Cas9 enables precise gene functional dissection through targeted knockout or knock-in of specific genes, allowing direct observation of the effects of gene loss or overexpression on fish phenotypes including development, behavior, and metabolism. Researchers demonstrated that targeted knockout of ehd3 in zebrafish resulted in a 2–3 fold significant increase in EPA/DHA/n-3 PUFA contents in muscle without affecting growth performance. This finding revealed the negative regulatory role of ehd3 in lipid homeostasis and provided a potential target for aquaculture nutritional improvement [75]. Furthermore, knockout of gluk2 in zebrafish larvae caused behavioral defects, reduced survival rates, and an approximately 60-fold increase in gill cell apoptosis under cold stress. Transcriptomic analysis revealed enrichment of temperature response pathways, confirming the critical role of gluk2 in fish cold tolerance behavior and stress metabolism [76]. Additionally, for in vivo imaging and dynamic cell tracking of the zebrafish midbrain-hindbrain boundary (MHB), researchers knocked in Venus/tRFP reporter genes at the otx2 and pax2a loci. These reporter genes were precisely controlled by endogenous promoters without interfering with endogenous gene function, enabling real-time cell tracking and in vivo imaging of MHB development and elucidating the regulatory roles of key genes in boundary formation during embryonic development [77]. Moreover, researchers developed a simple sequencing-free gRNA validation tool and an efficient CRISPR-Cas9 method that directly converted over 90% of injected embryos into F0 biallelic knockout individuals. This study demonstrated that CRISPR enables precise functional dissection from gene to behavioral phenotype within one week, making it suitable for high-throughput neurobehavioral studies [78].

CRISPR-Cas9 also enables simultaneous editing of multiple genes to investigate the regulatory mechanisms of gene interaction networks on fish traits, achieving precise dissection of complex regulatory networks through multiplex gene co-editing. Jao et al. [79] optimized the CRISPR/Cas system in zebrafish and demonstrated that simultaneous targeting of multiple genomic loci could generate additive multiple loss-of-function phenotypes in the same F0 generation fish, laying a technical foundation for subsequent studies on gene interaction networks and epistasis. Furthermore, Cai et al. [80] performed knockout of the *asb5a* and *asb5b* genes in zebrafish. Single knockout of *asb5a* resulted in no detectable phenotype, whereas double knockout of both genes caused cardiac dilation and abnormal heart rate. This study revealed the interaction mechanisms of 11 hub genes involved in cardiac contraction and precisely elucidated how functional redundancy and synergy regulate cardiac traits.

CRISPR-Cas9 also enables in-depth exploration of evolution and adaptation by validating key evolutionary loci through precise mutagenesis. Different fish species possess unique adaptive genes, providing natural experimental materials for investigating the interplay between natural selection and gene editing. The three-spined stickleback (*Gasterosteus aculeatus*) exhibits remarkable variation in the length of its prominent dorsal and pelvic spines among wild populations inhabiting different aquatic environments. Roberts Kingman et al. [81] used CRISPR/Cas9 to target exon 1 of the *Stc2a* gene and generate chimeric mutants in three-spined stickleback embryos. The results showed significantly increased lengths of dorsal spines 1/2 (DS1/DS2) and pelvic spines, providing causal evidence that *Stc2a* is a key evolutionary locus underlying the rapid adaptation of sticklebacks from marine to freshwater environments. Mexican cavefish (*Astyanax mexicanus*) have adapted to perpetual darkness through eye degeneration and the evolution of enhanced sensory systems, which also conserves energy. By comparing surface-dwelling fish and cavefish populations, Shennard et al. [82] used CRISPR/Cas9 to target the *rx3* exon and demonstrated that cis-regulatory variants at the *rx3* locus directly contribute to the evolution of eye size. This study represents the first validation of *rx3* as a critical evolutionary locus for eye degeneration in stable mutants, supporting its causal role in extreme environmental adaptation.

As summarized in Table 2, functional gene editing studies in fish show a clear bias toward the model organism zebrafish, which benefits from transparent embryos, high fecundity, short generation time, and well-established protocols. This enables rapid F0 screening [78] and high-throughput phenotyping of development, metabolism, stress response, and behavior. The asb5a/asb5b double gene knockout [80] enabled analysis of gene networks and regulatory elements. In contrast, applications in non-model evolutionary species such as three-spined stickleback and Mexican cavefish validate causal roles of specific loci in natural adaptation (spine length, eye degeneration), demonstrating the technology’s versatility beyond laboratory models. Common limitations across studies include mosaicism in F0 generations, variable germline transmission rates, and the need for species-specific optimization of gRNA design and delivery. Overall, CRISPR/Cas9 via embryo microinjection dominates, but the field is shifting toward precision tools (base/prime editing) and broader species coverage to accelerate both basic biology and translational applications (Everything in the table comes from what’s mentioned in the referenced articles, and “Not reported” means that part was not mentioned in the article).

### 3.2. Establishment of Fish Gene Editing Systems for Modeling Human Diseases

The application of gene editing technologies in modeling human cancer-causing mutations has also increased significantly. This is attributed to the maturation of gene editing tools such as cytosine base editors and CRISPR/Cas9, which enable the precise introduction of human cancer driver mutations to generate stable or transient cancer models. These models are subsequently utilized for high-throughput screening (HTS) to evaluate the efficacy, toxicity, and mechanisms of action of anti-cancer drugs. Yin et al. [83] constructed a knockout model of the *fbn1* gene in zebrafish to recapitulate the genetic defect underlying human Marfan syndrome. This study provided the first zebrafish platform for validation of mutation pathogenicity and mechanistic investigations of Marfan syndrome, supporting subsequent drug intervention testing. Furthermore, Wijerathna et al. [84] generated a larval knockout model of the *fech* gene in zebrafish to mimic erythropoietic protoporphyria 1 (EPP1). This model was directly applied for drug screening, offering an efficient platform for elucidating EPP1 pathogenesis and high-throughput drug development. Notably, Rosello et al. [85] developed a near-PAM-less cytosine base editor (CBE4max-SpRY) that enables efficient, PAM-unrestricted C-to-T point mutations and simultaneous multiplex gene editing, providing a powerful tool for investigating tumorigenesis mechanisms and screening personalized therapeutics. They further optimized multiple cytosine base editors to achieve precise C-to-T editing efficiencies of up to 91% without detectable indels or off-target effects. This optimized platform allows for the accurate introduction of human pathogenic SNVs, supporting the dissection of disease mechanisms including developmental disorders and cancer, as well as potential drug testing [55].

Table 3 highlights that human disease modeling in fish overwhelmingly relies on zebrafish due to its genetic conservation with humans and optical transparency, facilitating rapid validation of pathogenic SNVs and high-throughput drug screening. Traditional CRISPR/Cas9 knockouts generate loss-of-function models, while base editors (ABE/CBE) enable precise SNV modeling without DSBs, markedly reducing indels and enabling biallelic editing in F0/F1 generations. Applications have expanded from congenital disorders to cancer and developmental signaling pathways. Key advantages include visible phenotypes in larvae and cost-effective screening; however, limitations persist in off-target effects, mosaicism, and the need for stable germline lines for long-term mechanistic studies. Compared with functional genomics studies (Table 2), disease models increasingly incorporate precision editing tools, underscoring their translational value (Everything in the table comes from what’s mentioned in the referenced articles, and “Not reported” means that part was not mentioned in the article).

### 3.3. Aquaculture and Genetic Improvement

The core of traditional fish breeding consists of selective breeding and crossbreeding, which primarily rely on phenotypic observation and statistical approaches. Selective breeding for fast growth is the most intuitive and rapidly effective area in fish breeding, as growth traits exhibit relatively high heritability and are easily measurable. Mass selection is the most fundamental method, which involves selecting individuals with the largest body size and fastest growth rate as breeding parents from the same population based on indicators such as body weight and body length to produce the next generation. Significant improvements in growth rate can be achieved through 3–5 generations of consecutive selection. The most typical technique is family selection, which involves establishing multiple full-sib or half-sib families, rearing them under identical environmental conditions, selecting the families with the best growth performance, and then performing individual selection within these superior families.

Crossbreeding utilizes heterosis between different breeds or populations. By crossing breeds with fast growth but poor stress resistance with those with moderate growth but superior meat quality, the resulting F_1_ generation often exhibits heterosis in growth rate that exceeds the average of both parents. However, traditional breeding techniques have significant limitations, as factors such as inbreeding depression and environmental interference compromise their effectiveness.

Fish diseases represent a major challenge in aquaculture. Selective breeding for disease resistance is a difficult task in traditional breeding and also a key research priority in current aquaculture breeding programs. Disease resistance traits are characterized by low heritability and difficulty in direct measurement. Challenge testing is the core method for screening disease resistance traits. Since disease resistance cannot be directly observed under natural conditions, breeders artificially create infection challenges. Fish from different families are placed in the same water body containing pathogens, or artificially infected via injection or immersion, and the survival time and survival rate of each family or individual are recorded. Not all surviving fish are disease-resistant; some may be merely tolerant. Traditional breeding primarily uses these surviving individuals as broodstock candidates to establish families, as they carry resistance genes. For disease resistance traits, family selection is far superior to individual selection. Individual performance is strongly influenced by random factors. By establishing families and comparing their post-infection survival rates, antibody titers, or specific pathogen loads, families with high survival rates can be selected for propagation.

Gene editing technology has become a revolutionary tool for genetic improvement in aquaculture. Through precise targeting of genomic sequences to achieve knockout, knock-in, or base substitution, it enables targeted improvement of economic traits, including growth, disease resistance, and reproductive control. This technology overcomes the long-standing bottlenecks of traditional breeding, such as lengthy breeding cycles and low selection efficiency, providing strategic support for addressing climate change and frequent disease outbreaks, and holds significant economic, social, and ecological value. With the maturation of novel high-precision tools such as base editing and prime editing, combined with artificial intelligence (AI)-assisted gRNA design and multi-omics analysis, it is now possible to develop customized superior varieties that integrate multiple desirable traits: fast growth, high disease resistance, sterility, and superior quality. Controllable sterility technology represents the most promising approach to effectively mitigate ecological risks caused by the escape of gene-edited fish. However, it cannot completely eliminate all potential hazards, as existing sterility induction methods still face challenges including incomplete sterility rates, potential reversibility under specific environmental conditions, and limited applicability across different fish species. Overall, Gene editing has the potential to usher aquaculture into a new era of precision molecular breeding, offering unprecedented opportunities to accelerate genetic improvement of economically important traits, injecting strong impetus into the development of the Blue Granary and global sustainable aquaculture. However, its widespread industrial application still depends on the resolution of remaining technical challenges, the establishment of sound regulatory frameworks, the improvement of public acceptance, and the continuous improvement of animal welfare standards.

Editing of the myostatin (*mstn*) gene is a highly active research area in fish genetics. Myostatin functions as a negative regulator of muscle growth, and targeted knockout of this gene significantly promotes muscle development and increases body weight. Chinese researchers knocked out the *mstn* gene in grass carp (*Ctenopharyngodon idella*), achieving an editing efficiency of 67% in F0 individuals with substantial improvements in both meat quality and production yield [86]. Japanese researchers knocked out *mstn* in red sea bream (*Pagrus major*) [87], resulting in increased muscle mass and an over 20% faster growth rate, which enhanced its edible value and has already been commercialized. Knockout of *runx2b* and *bmp6* has been used to develop intermuscular bone-free blunt snout bream (*Megalobrama amblycephala*) [88] and common carp (*Cyprinus carpio*) [89], thereby improving their edible and processing values. Editing of immune genes such as *gcJAM-A27* [90], *irf3* [91], *tnf-α1* [92], and *SOCS3a/3b* [93] enhances antiviral and antibacterial capabilities and reduces antibiotic usage. Pavelin et al. [94] combined QTL mapping with CRISPR/Cas9 to knock out the *nae1* gene in Atlantic salmon (*Salmo salar*) cell lines, confirming that *nae1* is the major causal gene controlling resistance to infectious pancreatic necrosis virus (IPNV). Notably, Coogan et al. [95] used CRISPR/Cas9 to knock out the *mstn* gene in channel catfish (*Ictalurus punctatus*) embryos. The resulting mutants exhibited significantly greater body weight and length than wild-type fish at all growth stages. In bacterial challenge tests, the mutants showed increased survival rates and prolonged mean time to death. This study represents the first demonstration that *mstn* knockout can simultaneously achieve rapid growth and enhanced bacterial resistance, providing direct evidence for single-gene multiple-effect disease-resistant breeding in aquaculture.

**Figure 3:**
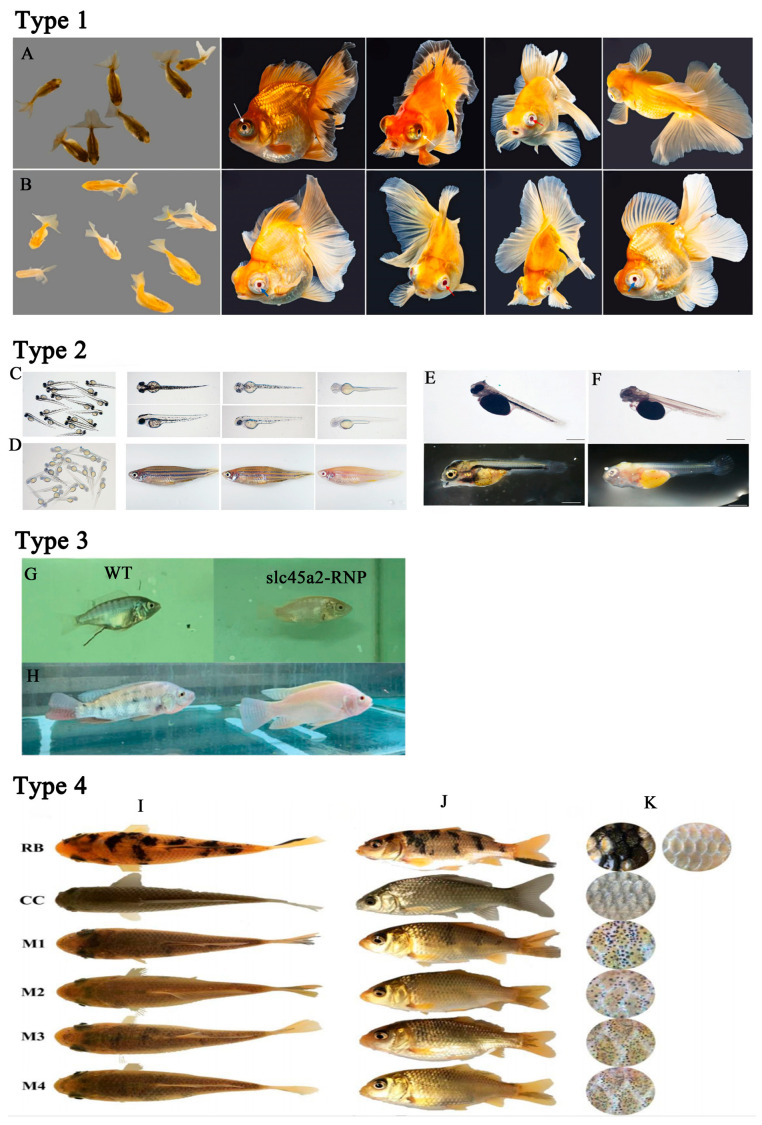
Type 1: (**A**) represents wild-type goldfish, while (**B**) depicts goldfish with *tyr* knockout-induced albino mutations, demonstrating that *tyr* disruption results in the loss of melanophores [96]. Type 2: Left panels (**C**,**D**) show zebrafish at various developmental stages exhibiting albinism due to *slc45a2* knockout; (**E**,**F**) displays wild-type and *slc45a2* knockout-derived albino juveniles of the redhead cichlid (*Vieja melanura*) [97]. Type 3: (**G**) shows a wild-type Nile tilapia (*Oreochromis niloticus*), and (**H**) shows an *slc45a2*-knockout Nile tilapia. Knockout of the *slc45a2* gene in this species results in uniform red body coloration throughout the entire body [98]. Type 4: RB denotes wild-type Oujiang color common carp (*Cyprinus carpio*) with large black spots, CC denotes wild-type common carp, and M1–M4 represent Oujiang color common carp subjected to targeted disruption of *asip* [100]. (**I**) Top view of fish of types RB, CC, and M1–M4; (**J**) Side view of each type of fish; (**K**) Skin details of each type of fish.

Currently, the global ornamental fish aquaculture industry is developing rapidly, and vibrantly colored ornamental fish are highly favored by consumers. Studies have shown that gene editing for breeding ornamental fish with unique color patterns by targeting pigment-related genes allows for the observation of distinct phenotypic changes as early as the F0 generation, and these traits can be stably inherited to establish pure lines. Next-generation gene editing tools such as base editing and prime editing can reduce off-target probabilities and enable more precise establishment of stable lines, thereby facilitating large-scale commercial production. Elucidating the developmental mechanisms of pigment cells through scientific research is of great significance for reducing wild harvesting, enriching consumer choices, and advancing vertebrate evolution research. Specifically, knockout of the *tyr* gene [96] results in complete loss of melanocytes, producing golden goldfish with bright golden body color and red eyes, and creating a novel golden ornamental goldfish line. Knockout of *slc45a2* [97,98] can generate albino or uniform red lines, creating significant ornamental and commercial value. Editing of the *alkal2l* gene [99] modulates the blue/red color ratio in the scales and skin of Siamese fighting fish (*Betta splendens*). Disruption of the *asip* gene [100] disperses or redistributes black spots in Oujiang color common carp (*Cyprinus carpio*), forming novel red-black patterns. Representative phenotypic images of these gene-edited ornamental fish are summarized in Figure 3.

Table 4 provides a comparative overview of gene editing applications in aquaculture breeding and ornamental fish. CRISPR/Cas9 microinjection in embryos remains the dominant platform, achieving high F0 editing efficiencies and visible phenotypes suitable for rapid selection. Growth-related targets consistently yield muscle hypertrophy and faster growth across freshwater and marine species, with some studies demonstrating pleiotropic benefits, growth, and disease resistance in channel catfish [95]. Intermuscular bone elimination and immune gene knockouts address key industry pain points (processing quality and antibiotic reduction). Ornamental applications target pigment genes (*tyr*, *slc45a2*, *asip*, *alkal2l*), producing striking color/pattern changes often detectable in F0 and stably heritable, enabling commercial line development with minimal generations. Compared with functional/disease studies (Table 2 and Table 3), aquaculture applications emphasize economic traits and biosafety, yet face greater challenges in large-egg marine species, germline transmission rates, and regulatory approval. Base/prime editing is still underrepresented in breeding programs but holds promise for minimizing off-targets and enabling multi-trait stacking (Everything in the table comes from what’s mentioned in the referenced articles, and “Not reported” means that part was not mentioned in the article).

## 4. Ethical and Ecological Safety Considerations

### 4.1. Environmental Risk Assessment of Genome-Edited Fish

Gene editing technologies, particularly CRISPR/Cas9 and its derivative tools, enable precise improvement of economic traits such as growth, disease resistance, and sterility in aquaculture. However, their widespread application has simultaneously raised profound ethical, ecological, and environmental safety concerns. These concerns encompass animal welfare, consumer right to know and food safety transparency, social equity, and philosophical debates over the deliberate alteration of natural evolutionary processes. Ecologically, the primary risk is gene flow: escaped gene-edited fish may hybridize with wild conspecifics, leading to introgression—the transfer of exogenous or edited alleles into wild genomes. This can alter the adaptive fitness of wild populations, reduce their genetic diversity [12], and even trigger biological invasions. In the long term, gene-edited fish may outcompete their wild counterparts in survival, reproductive capacity, disease resistance, or behavioral traits, disrupting food webs [101] and gaining a competitive advantage in ecosystems. This could result in the decline of wild populations, alteration of ecosystem structure, or functional impairment.

Effective ecological risk prevention can be achieved through a comprehensive assessment of genetic contamination from gene flow, ecological changes induced by off-target effects, and the impacts of escaped fish on food webs and biodiversity. To mitigate the ecological impacts of escaped fish and the spread of modified traits to wild populations [102], several mitigation strategies have been proposed: gene editing can be used to induce sterility, such as knocking out the *dnd* gene to generate sterile lines that effectively prevent genetic contamination from escaped Atlantic salmon (*Salmo salar*) [103]; multi-omics monitoring and ecological modeling have also been advocated to predict food web perturbations [104].

The core environmental risk of gene-edited fish lies in irreversible gene spread and niche disruption, rather than acute toxicity or pathogenicity. The most effective countermeasure is not post-escape recapture, but proactive reproductive containment that ensures edited fish cannot reproduce in natural environments. This must be complemented by multi-layered physical containment, real-time monitoring, and rapid eradication capabilities to control risks within acceptable limits. If a gene-edited fish cannot be 100% confirmed to be reproductively sterile after escape, it should not be permitted to enter any environment connected to natural water bodies.

### 4.2. Additional Risks, Ethical and Social Considerations of Gene-Edited Fish

In addition to the ecological risks caused by gene flow and wild population introgression, a rigorous safety evaluation of gene-edited fish must systematically address multiple layers of technical, biological, ethical, and practical challenges. From a technical perspective, off-target cleavage and associated structural genomic variants represent the most direct unintended outcomes of editing; at the organismal level, founder chimerism and unexpected pleiotropic fitness trade-offs affect trait stability and environmental adaptability; in terms of application and governance, animal welfare, post-escape monitoring efficacy, consumer acceptance, and inherent limitations of captive breeding systems jointly determine the feasibility of industrialization. A comprehensive analysis of these interrelated risk dimensions is essential to establish a sound risk mitigation system and advance the responsible deployment of gene editing in aquaculture.

In gene-edited fish models, off-target effects refer to the non-specific cleavage of DNA by CRISPR-Cas9 at loci outside the intended target site, which may generate insertions/deletions (indels) or large structural variants (SVs). In a landmark study by Höijer et al. [105], fertilized zebrafish eggs were edited with four guide RNAs, and long-read sequencing analysis of DNA from over 1100 larvae, juveniles, and adult fish across two generations revealed that SVs occurred at both on-target and off-target editing sites. Additionally, germ cell mosaicism was observed in adult founder zebrafish. Therefore, in aquaculture, off-target effects may cause unpredictable health or growth defects, thereby amplifying the commercialization risks of gene-edited aquatic organisms.

Chimerism is also extremely prevalent following CRISPR microinjection into fish embryos, as Cas9 activity can persist into the multicellular stage, resulting in genotypic heterogeneity across different cell lineages. The introduction of specific edited genes may also be accompanied by detrimental effects on other traits due to pleiotropy. Gratacap et al. [14] noted in a comprehensive review of aquatic gene editing that founder generation (F0) chimerism represents a major technical barrier, which may lead to germline instability and prevent reliable transmission of edited alleles to subsequent generations. Moreover, the rapid embryonic development of fish results in a significantly higher incidence of chimerism compared to mammals. In aquaculture applications, chimerism not only hinders the establishment of stable breeding lines but also produces fish populations with inconsistent phenotypes, increasing the difficulty of standardized animal welfare assessment. Additionally, edits designed to enhance growth may induce pleiotropic perturbations and impose overall fitness costs, including increased disease susceptibility and reduced predator avoidance ability in natural environments. Against the backdrop of high escape rates of farmed fish, adaptive trade-offs are particularly critical: traits optimized for intensive aquaculture may reduce wild competitiveness, leading to population collapse following accidental release into natural ecosystems.

Unexpected pleiotropic effects refer to the phenomenon where a single gene edit affects multiple biological pathways or phenotypic traits. In a comprehensive review on sustainable CRISPR applications in fish aquaculture, Okoli et al. [104] explicitly pointed out that pleiotropic effects may still occur even with precise on-target editing, particularly in the modification of complex traits, necessitating comprehensive phenotypic screening. Fish genomes frequently contain duplicated genes, which further amplifies the risk of pleiotropy. Neglecting long-term multi-generational monitoring will lead to underestimation of fitness losses in both aquaculture settings and natural environments, especially in commercial editing programs aimed at enhancing growth performance.

Gene editing can improve fish welfare by enhancing disease resistance or editing sterility genes, but the microinjection procedure and unexpected phenotypes may cause animal suffering. Cage escape events are frequent in aquaculture, and post-escape monitoring of gene flow, phenotypic stability, and ecological feedback is mandatory for gene-edited fish. Consumer acceptance of gene-edited fish is generally lower than that of gene-edited plants, influenced by concerns over animal welfare, food safety, and ecological risks. Robinson et al. [106] proposed a dedicated assessment framework for aquatic gene editing, which lists animal welfare as one of the nine core considerations. The framework emphasizes the need to evaluate editing impacts on fish behavior, physiological health, and natural integrity; identifies environmental DNA (eDNA)-based monitoring as a critical tool for detecting escaped individuals; and notes that the dnd gene-edited sterility strategy can mitigate gene flow risks, although long-term field monitoring is still required to confirm its irreversibility. Public acceptance is explicitly highlighted as the decisive factor for large-scale commercialization: acceptance increases significantly when edits deliver measurable welfare improvements, environmental benefits, or enhanced food safety, whereas edits targeting solely production traits face widespread public resistance.

Fish gene editing is highly dependent on hatchery/aquaculture broodstock breeding, yet it faces classic limitations, including genetic drift, domestication selection, and reintroduction failure. Snyder et al. [107] noted in an early review that captive environments lead to loss of adaptation and low post-escape survival rates, while high operational costs, disease outbreaks, and genetic bottlenecks remain prominent challenges. Although the high fecundity of fish facilitates scalability, selection pressures in captive environments amplify the phenotypic instability of edited traits. Even though CRISPR can rapidly restore genetic diversity, captive breeding bottlenecks still restrict genetic representation and wild adaptation potential.

The widespread and responsible application of gene-edited fish will become a reality only when a comprehensive risk mitigation framework is fully implemented: off-target effects are controlled by high-fidelity Cas9 variants and whole-genome validation; chimerism is minimized through optimized delivery systems; pleiotropic risks are addressed via multi-generational phenotypic screening; animal welfare is embedded in all stages of research and development; adaptive trade-offs are quantified through rigorous fitness assays; post-escape monitoring is established using eDNA technology; public trust is built through transparent communication; and captive breeding limitations are overcome by integrating conservation genetics principles.

### 4.3. International Regulatory and Policy Landscape

In fish, transgenesis, gene editing, SDN-1, SDN-2/SDN-3, and CRISPR editing represent distinct technical concepts at different hierarchical levels. Transgenesis falls under classical genetic modification (GMO), characterized by the insertion of exogenous DNA. Gene editing is the broad umbrella term for targeted genome modification [108]. SDN-1, SDN-2, and SDN-3 are classifications of outcomes generated by site-directed nucleases (SDNs) according to regulatory frameworks such as those established by the European Food Safety Authority (EFSA) [109]. CRISPR editing, as the most widely used specific tool currently, is capable of generating all three SDN types of edits.

Regulatory frameworks for gene-edited aquatic animals vary significantly across countries. The United Nations and international organizations [110] mandate environmental and ecological risk assessments for genetically modified and gene-edited organisms prior to release, regulate cross-border transfers, and protect biodiversity. The Food and Agriculture Organization (FAO) and World Health Organization (WHO) [111] have developed risk assessment guidelines for gene editing technologies in aquatic animals, emphasizing scientific evaluation, transparent governance, and public participation.

In China, the Ministry of Agriculture and Rural Affairs [112] has established regulations governing environmental risk, food safety, and industrialization assessments of gene-edited fish, incorporating them into the Regulations on the Administration of Agricultural Biosafety. China actively promotes research and development while emphasizing controllable scope and biosafety, and has not yet approved large-scale commercialization of gene-edited aquatic products. The United States [113] regulates gene-edited animals as new animal drugs, requiring safety and ecological impact assessments. Notably, AquAdvantage salmon—the only FDA-approved genetically engineered salmon—was developed using classical transgenic technology rather than CRISPR-mediated gene editing. The European Union [114,115] adopts a highly cautious approach toward gene-edited animals and Plants, with low public acceptance. The EU classifies all gene editing technologies as genetically modified organisms (GMOs), requiring strict regulatory approval even for SDN-1 type edits. Argentina [116] has taken a more innovation-friendly stance: it does not consider SDN-1 edits as GMOs if they contain no exogenous DNA and have no significant unintended effects. Argentina promotes commercialization while balancing innovation with biosafety, facilitating faster market entry for gene-edited products. Japan [117] similarly does not classify SDN-1 gene-edited organisms as GMOs, although safety assessments are still required for food applications.

## 5. Conclusions

Fish gene editing technologies have evolved from specialized laboratory tools into foundational tools that underpin breakthroughs in basic biology and support transformative advances in the aquaculture industry. From zinc-finger nucleases (ZFNs) and transcription activator-like effector nucleases (TALENs) to CRISPR/Cas9 and its high-precision derivative platforms, a closed-loop strategy encompassing target trait identification, causal locus validation, controllable editing, multi-omics evaluation, and biosafety and compliance considerations is poised to become the central framework for the sustainable development of fish gene editing research and application.

Gene editing technology holds substantial scientific and practical value for fish research. It not only accelerates basic biological discovery but also supports the sustainable development of aquaculture. This technology has advanced our understanding of genotype-phenotype relationships from statistical correlation to reproducible causal evidence, and shifted the paradigm of traditional breeding from phenotypic screening to precision molecular design. It has markedly shortened breeding cycles and enabled targeted improvement of economically important traits. Gene editing tools have also expanded the scope of basic fish research, allowing aquaculture scientists to precisely manipulate genetic material and dissect complex physiological mechanisms.

Beyond its role as a research tool, gene editing technology represents a key enabler for the transformation of aquaculture from a resource-dependent model to a technology-driven one, deepening scientific insights into fish biology and underpinning the high-quality development of the industry. Meanwhile, a balance must be struck between innovation and safety to provide strategic support for the construction of the Blue Granary. Only by achieving a verifiable balance between innovation-driven development and ecological protection can fish gene editing truly become a green revolutionary force promoting high-quality aquaculture development, ensuring food security, and conserving biodiversity. In the future, more genetically improved high-performance fish varieties will appear on dining tables worldwide, providing humans with higher-quality and sustainable protein sources.

## Figures and Tables

**Table 1:** Comparison of Characteristics among Different Gene Editing Platforms and Derivative Technologies.

Platform/Technology	Mechanism of Action	Key Advantages	Key Limitations	Corresponding References
ZFN	Protein recognition and FokI cleavage	Early achievement of site-directed DSB induction	Intricate design, high cost, and limited scalability	[35,36,37]
TALEN	Repeat module recognition and FokI cleavage	Well-defined targeting rules, superior specificity	Large protein size, substantial construction, and delivery burden	[36,38,39]
CRISPR/Cas9	gRNA-directed Cas cleavage generates DSB	Simple design, high efficiency, multiplex editing capability	Relatively high off-target activity, PAM sequence dependence	[31,36,40]
Base editing	Deaminase-catalyzed single-nucleotide conversion; DSB-independent	Reduced indels; suitable for modeling SNVs, functional validation of disease-associated variants, and refined trait modification	Constrained editing window and bystander edits; limited targetable sites	[41,42,43,44]
Prime editing	Reverse transcription-mediated editing; DSB- and donor DNA-independent	Enables diverse, precise editing outcomes and short fragment insertion/repair	Efficiency influenced by system composition and delivery; complex design; application at loci recalcitrant to HDR-mediated modification	[45,46,47]
dCas9-mediated regulation	Cleavage-independent, targeted modulation of transcription and/or epigenetic states	No alteration of DNA sequence; reversible regulation; suitable for mechanistic dissection, environmental response, and plasticity studies	Optimization required for modulation strength and persistence	[48,49,50,51]

**Table 2:** Summary of Gene Editing Applications in Fish Functional Genomics and Evolutionary Studies.

Fish Species	Target Gene(s)	Editing Platform	Delivery Method	Editing Efficiency	Phenotype	Application Stage	Key Limitations
Zebrafish [75]	*ehd3*	CRISPR/Cas9	Microinjection	Not reported	↑ EPA/DHA/n-3 PUFA in muscle; no growth impact	Embryo → F0 juveniles/adults	Mosaicism common; ideal for rapid lipid trait screening
Zebrafish [76]	*gluk2*	CRISPR/Cas9	Microinjection	Not reported	Behavioral defects, ↓ survival, ↑ gill apoptosis under cold stress	Not reported	Not reported
Zebrafish [77]	*otx2*, *pax2a*	CRISPR/Cas9 knock-in	Microinjection	Successful	Reporter expression for MHB imaging and cell tracking	Not reported	Not reported
Zebrafish [78]	*Various* (*behavior*)	CRISPR/Cas9	Microinjection	>90% biallelic in F0	Rapid behavioral phenotypes	F0 (one-week phenotyping)	Excellent for high-throughput; germline validation recommended for stable lines
Zebrafish [80]	*asb5a/asb5b*	CRISPR/Cas9 (double KO)	Microinjection	Successful double KO	Cardiac dilation and abnormal heart rate (single KO no phenotype)	Not reported	Not reported
Three-spined stickleback [81]	*Stc2a*	CRISPR/Cas9	Microinjection	Not reported	Increased dorsal/pelvic spine length	Embryos, chimeric mutants	Non-model species; causal evolutionary validation achieved
Mexican cavefish [82]	*rx3*	CRISPR/Cas9	Microinjection	Successful	Validation of eye size degeneration	Embryos to adults	First causal validation in stable lines

**Table 3:** Summary of Gene Editing Applications for Human Disease Modeling in Fish.

Fish Species	Target Gene(s)	Editing Platform	Delivery Method	Editing Efficiency	Phenotype	Application Stage	Key Limitations
Zebrafish [83]	*fbn1*	CRISPR/Cas9	Microinjection	Not reported	Marfan syndrome model	Not reported	Not reported
Zebrafish [84]	*fech*	CRISPR/Cas9	Microinjection	Not reported	Erythropoietic protoporphyria 1 (EPP1); drug screening	Not reported	Not reported
Zebrafish [52]	*dapk3*, *ube2b*, *usp44*, *ptpn11*	Adenine/cytosine base editors	Microinjection	Efficient biallelic	Congenital heart disease SNV modeling	Embryo microinjection, F0/F1	Excellent for SNV modeling; minimal indels
Zebrafish [53]	*Various*	ABE-ultramax	Microinjection/RNP	High biallelic	Developmental signaling and human pathology	Not reported	Not reported
Atlantic salmon [54]	*Various*	AncBE4max (C-to-T)	Embryo injection	Efficient C-to-T	Disease resistance potential	Embryo injection	Aquaculture-relevant; scalable to breeding programs
Zebrafish [55,85]	*ctnnb1*, *cbl*	CBE4max-SpRY/near-PAM-less	Microinjection/RNP	Up to 91% (no indels)	Wnt activation, cancer, dwarfism models	F0/F1 + stable lines	Near-PAM-less; powerful for precise human mutation modeling

**Table 4:** Summary of Gene Editing Applications in Aquaculture Breeding and Ornamental Fish Traits.

Fish Species	Target Gene(s)	Editing Platform	Delivery Method	Editing Efficiency	Phenotype	Application Stage	Key Limitations
Grass carp [86]	*mstn b*	CRISPR/Cas9	Microinjection	67% in F0	Muscle hypertrophy, improved growth/meat quality	Embryo microinj., F0 juveniles (7 mo)	Mosaicism common; strong candidate for meat-yield breeding but needs germline validation and biosafety.
Red sea bream [87]	*mstn*	CRISPR/Cas9	Microinjection	High	>20% faster growth, ↑ muscle mass	F0 founders → F1/F2 juveniles (commercial line)	Demonstrates scalability potential.
Channel catfish [95]	*mstn*	CRISPR/Cas9	Microinjection	Not reported	↑ body weight/length and bacterial resistance	Embryos → F0 + F1 multi-stage growth/challenge	First single-gene multi-effect (growth and disease resistance).
Blunt snout bream [88]	*runx2b*	CRISPR/Cas9	Microinjection	Not reported	Intermuscular bone-free	F0 screening → stable lines	Pleiotropy monitoring needed.
Common carp [89]	*bmp6*	CRISPR/Cas9	Microinjection	Not reported	Intermuscular bone-free	Multi-generational strain development	Time required for homozygosity.
Grass carp/zebrafish [90,91,92,93]	*gcJAM-A*, *irf3*, *tnf-α1*, *SOCS3*	CRISPR/Cas9	Microinjection	Variable	Enhanced antiviral/antibacterial resistance	Not reported	Not reported.
Atlantic salmon (cell line) [94]	*nae1*	CRISPR/Cas9	Lentiviral	Not reported	IPNV resistance	Not reported	Not reported.
Goldfish [96]	*tyr*	CRISPR/Cas9	Microinjection	High	Golden body, red eyes (albino)	Embryo F0 + stable ornamental lines	Rapid F0 phenotype ideal for ornamental breeding; low off-target with precision tools.
Zebrafish, redhead cichlid, Nile tilapia [97,98]	*slc45a2*	CRISPR/Cas9	Microinjection	High	Albinism or uniform red coloration	F0 visible → F1 stable pure lines	High ornamental/aquaculture commercial value; visual marker for efficiency.
Siamese fighting fish [99]	*alkal2l*	CRISPR/Cas9	Microinjection	Not reported	Modulation of blue/red color ratio	Embryo F0	Pattern modulation; environment × genotype interactions possible.
Oujiang color common carp [100]	*asip*	CRISPR/Cas9	Microinjection	Successful	Black spot dispersion → novel red-black patterns	F0 + heritable lines	Striking visual novelty.

## Data Availability

No new data were created or analyzed in this study. Data sharing is not applicable to this review.
